# Synthesis and Antibacterial Assessment of *N*-[4-(4-substituted phenyl)-1,3-thiazol-2-yl]-1,3,5-triazin-2-amine

**DOI:** 10.4103/0250-474X.51960

**Published:** 2009

**Authors:** P. Gahtori, A. Das, H. Bhatt

**Affiliations:** Department of Pharmacy, Kumaun University, Bhimtal-263 136, India; 1 Department of Pharmaceutical Sciences, Dibrugarh University, Dibrugarh-786 004, India

**Keywords:** Bacterial resistance, physicochemical properties, s-Triazine, broth dilution technique, antibacterial activity

## Abstract

In a wide search program towards new and efficient antibacterial agents, we assessed the extent to which physicochemical properties can be exploited to promote antibacterial activity associated with a series of substituted s-triazine. The synthesized compounds (1a-12b) were subsequently screened for their *in vitro* antibacterial activity against three gram positive (*Bacillus subtilis, Bacillus cereus, Staphylococcus aureus*) and three Gram-negative microorganism (*Salmonella typhi, Escherichia coli, Klebsiella aerogenes*) by the broth dilution technique, recommended by European Committee for antimicrobial susceptibility testing with reference to streptomycin.

The extraordinary progress represented by the arrival of antibiotics has changed the medical prognosis of minor and major infections. However, over the years via several constantly changing mechanisms, many bacterial species acquired resistance to the most common classes of antibiotics. A relevant report on resistant antibacterial agents for human medicine is provided by World Health Organization. The panel agreed that the list of critically important antibacterial agents should be updated regularly as new information becomes available, including data on resistance patterns, new and emerging diseases and the development of new drugs[1].

During the last few years the potential of s-triazine derivatives in molecular recognition, agrochemical and medicinal properties have been subjected to investigation. Literature survey reveals that substituted s-triazine derivatives are associated with number of pronounced antibacterial activities[2–4] against gram positive (*B. subtilis, B. cereus* and *S. aureus*) and gram negative organism (*S. typhi, E. coli* and *K. aerogenes*). The biological activity is a function of physicochemical properties of the targeted molecule and this assessment is made of the sorts of chemicals that might fit into an active site[5,6]. To randomly explore the novel compounds, our idea was to combine, cyanuric chloride with various amines possessing distinguish physicochemical properties. The substituted phenylthiazole-2-amine derivatives remain attractive, with their significant biological activities[7,8] and further incorporation of these derivatives giving access to a wide array of structures, which can be expected to show interesting antibacterial activities. In this wide search program, here we demonstrate various amines mediated substitution in cyanuric chloride and subsequently with higher bioactive, 4-sustituted phenylthiazole-2-amine to synthesize *N* -[4-(4-substituted phenyl)-1,3-thiazol-2-yl]-1,3,5-triazin-2-amine derivatives (scheme 1).

**Scheme 1:**
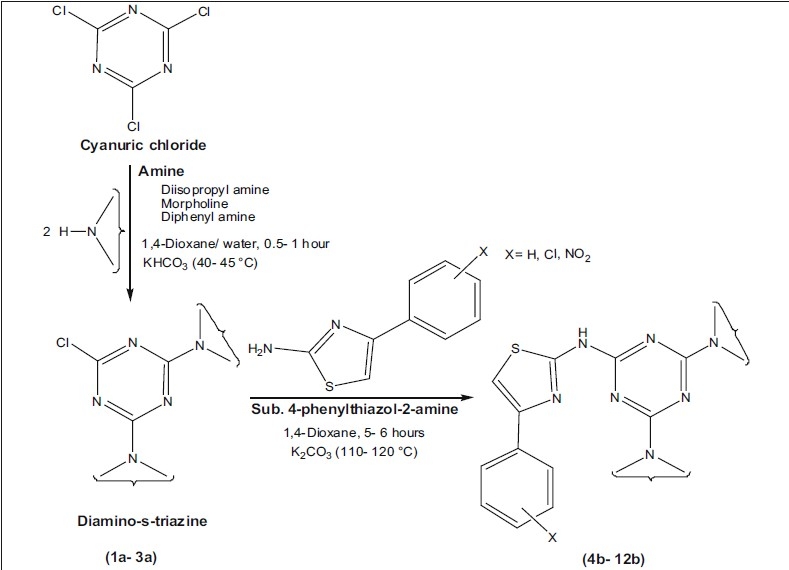
Synthesis of N-[4-(4-substitutedphenyl)-1,3-thiazol-2-yl]-1,3,5-triazin-2-amine (4b-12b)

Phenyl, 4-chlorophenyl and 4-nitrophenyl thiazole-2-amine derivatives were synthesized according to literature procedures[9,10], with the help of corresponding acetophenone, thiourea, thionyl chloride and bromine. The designed compounds were prepared in two step reactions, first step consists of nucleophilic substitution of two chlorines in cyanuric chloride in presence of aq. dioxane[11] with various amines like diisopropylamine, morpholine and diphenylamine to synthesize (1a-3a) diamino-s-triazines (0.5-1 h) and second step involves further substitution of third chlorine in presence of 1,4-dioxane[12] with synthesized thiazole-2-amine to obtain a series (4b-12b) of *N*-[4-(4-substituted phenyl)-1,3-thiazol-2-yl]-1,3,5-triazin-2-amine (5-6 h).

Subsequently synthesized compounds were screened for their *in vitro* antibacterial activity (MIC) against three gram positive (*Bacillus subtilis, Bacillus cereus, Staphylococcus aureus)* and three gram negative microorganism (*Salmonella typhi, Escherichia coli, Klebsiella aerogenes*) by the broth dilution technique, recommended by European Committee for antimicrobial susceptibility testing (EUCAST) E. Dis 5.1[13] with reference to streptomycin. Nutrient agar (M090) and nutrient broth (M002) were procured from Himedia Laboratories, Mumbai. A set of sterilized test tubes with nutrient broth medium capped with cotton plugs (1–8). The test compound is dissolved in dimethylsulfoxide (512 μg/ml), which are serially diluted from 1 to 8. One millilitre of the standardized broth culture was added to 1 ml of each serially diluted test tube. The capped tubes with cotton plugs were incubated at 35-37° for 20 h and compared with standardized 0.5 McFarland reagent.

The MIC values (Table 1) of this class of synthesized compounds against tested organism displayed a significant activity with wide degree of variation (4-128 μg/ml). The preliminary antibacterial results showed that diamino-s-triazine (1a-3a) derivatives were significantly less active, among them diphenylamine substituted s-triazine were more efficient substituent for the entire tested microorganisms. Further a considerable difference in antibacterial activity between (1a-3a) disubstituted, diamino-s-triazine (MIC= 16-128 μg/ml) and (4b-12b) trisubstituted, *N*-[4-(4-substitutedphenyl)-1,3-thiazol-2-yl]-1,3,5-triazin-2-amine (MIC= 4-64 μg/ml), emphasized the importance of incorporating bulky groups. All of the biologically active substituted s-triazine derivatives show lipophilic traits. Possibly, this is because of the enhanced ability of compound to penetrate the cell permeability barrier. The substituted s-triazine derivatives (6b, 9b and 12b have shown significant antibacterial activity, suggesting that the 4-nitrophenyl thiazole-2-amino group was the all-important functional moiety (fig. 1).

**TABLE 1:** *IN VITRO* ANTIBACTERIAL ACTIVITY OF THE SYNTHESIZED COMPOUNDS

Compound	MIC (μg/ml)					
	
	*B. subtilis*	*B. cereus*	*S. aureus*	*S. typhi*	*E. coli*	*K. aerogenes*
1a	64	128	64	32	16	32
2a	128	128	128	128	64	128
3a	32	64	64	64	16	64
4b	16	16	16	32	16	8
5b	32	64	16	32	16	16
6b	8	8	8	16	8	32
7b	64	16	16	64	16	32
8b	4	16	16	16	4	32
9b	8	8	16	16	8	8
10b	16	32	4	32	8	32
11b	32	8	32	8	16	16
12b	4	8	8	16	4	32
Streptomycin	8	4	8	< 2	< 2	< 2

*DMSO as negative control

**Fig. 1:**
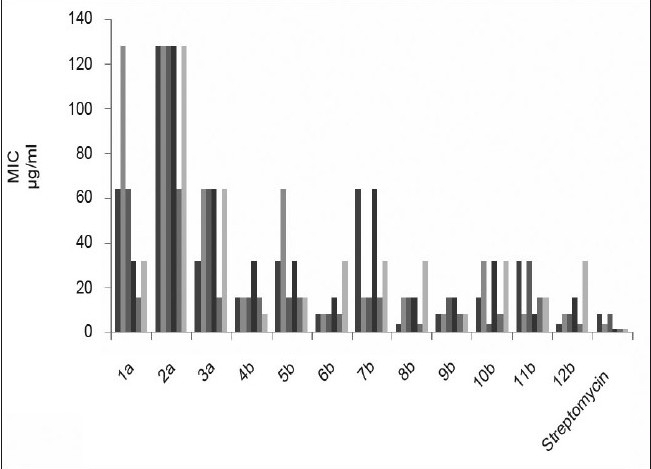
Antibacterial results against Streptomycin as standard control MIC values for all tested microorganisms, 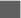 *B. subtilis*, 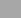 *B. cereus*, 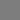  *S. aureus* 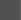 *S. typhi*, 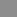 *E. coli*, 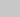 *K. aerogenes*

Today up to 75% of antibiotic use is said to be of questionable therapeutic value, antibiotic use is the key driver of resistance. We have presented a new economical synthesis of *N*-[4-(4-substitutedphenyl)-1,3-thiazol-2-yl]-1,3,5-triazin-2-amine. The operational simplicity, significant short reaction times and relevant antibacterial results can impose this procedure as a useful and attractive alternative.
